# Advances in MS-Based Analytical Methods: Innovations and Future Trends

**DOI:** 10.1155/2018/2084567

**Published:** 2018-10-08

**Authors:** Federica Bianchi, Leopold Ilag, Veronica Termopoli, Lucia Mendez

**Affiliations:** ^1^Department of Chemistry, Life Sciences and Environmental Sustainability, Parma University, Parco Area delle Scienze 17/A, 43124 Parma, Italy; ^2^Department of Environmental Science and Analytical Chemistry, Stockholm University, 10691 Stockholm, Sweden; ^3^Department of Pure and Applied Sciences, LC-MS Laboratory, Piazza Rinascimento 6, 61029 Urbino, Italy; ^4^Instituto de Investigaciones Marinas, Spanish National Research Council (IIM-CSIC), Eduardo Cabello, 6, E-36208 Vigo, Spain

Mass spectrometry (MS) is a widely used technique for food safety, environmental, pharmaceutical, biological, and forensic investigations where the simultaneous detection of targeted and nontargeted compounds is of pivotal importance. A plethora of analytical MS methods also coupled to different separation techniques such as gas- and liquid-chromatography and their multidimensional analogues or capillary electrophoresis have been developed and validated in order to analyze complex matrices. However, despite the rapid evolution from its beginning, the development of online and real-time analytical MS methodologies especially ambient ionization methods is strongly demanded to perform high-throughput analysis and to obtain highly informative spectra. In this context, novel materials and instrumental configurations are under study to enhance the performance of the different instruments, whereas powerful high-resolution mass spectrometers are required to univocally identify targeted compounds. Finally, libraries of compounds, including MS-based information such as accurate mass, isotopic patterns, and collision-induced fragmentation, are strongly demanded together with studies regarding the establishment of recognized analytical performance criteria to assess the occurrence of residues in the environment. Mass spectrometric imaging is another emerging powerful analytical technique that can be applied to perform analyses of multiple molecules in complex samples without labeling, thus providing a distinct advantage over preexisting methods for label-free and simultaneous detection of drugs and metabolites.

This special issue covers the broad area of MS-based analytical methods starting from the development of novel LC-MS and LC-MS/MS methods for the quantitation of compounds of environmental and pharmaceutical concern to the use of more advanced separation technologies coupled to mass spectrometry for the analysis of atmospheric samples. More precisely, F. Bianchi et al. have provided a discussion of some instrumental innovations and their applications in the field of mass spectrometry with particular attention to spray-based MS methods and LC-EI-based MS interfaces. New materials, prototypes, and instrumental configurations able to increase the performance of the developed methods are presented and discussed. Finally, an overview of the most recent MS-based methods in food analysis is given covering the state of the art from 2012 up to 2017. L. F. Angeles and D. S. Aga have described the role of the ion ratio in the reconnaissance of pharmaceutical compounds in aquatic environment using LC-MS. Establishing performance criteria for the global reconnaissance of pharmaceuticals is important since it minimizes the occurrence of false-positive and false-negative detection. Based on these assumptions, a performance criterion was disclosed by the authors and applied to several equal-to-real samples. For environmental assessment, in situ radiogenic isotope determinations with microscale resolution can represent a powerful tool especially for geological and life sciences: S. Di Salvo et al. have presented a detailed methodological description of the analytical procedure from sampling to elemental purification and Sr-isotope measurements. The proposed method offers the potential to attain isotope data at the microscale on a wide range of solid materials with the use of minimally invasive sampling.

Mass spectrometry plays a pivotal role also in the forensic field: in this context, N. Mogollon and coworkers have reviewed the recent developments in MS for forensic analysis focusing their attention to the identification and quantification of drugs of abuse in biological fluids, tissues, and synthetic samples. Both the most common methodologies and the new methodologies used for screening and target forensic analyses are reviewed, thus including high-resolution MS as well as the use of ambient ionization ion sources for high throughput and real-time monitoring.

When very complex matrices have to be analyzed, multidimensional chromatography coupled to mass spectrometry can offer increased selectivity and separation power to solve different analytical problems: Y. G. Ahn et al. compared the performances of gas chromatography with quadrupole mass spectrometry and GC × GC-TOFMS for quantitative analysis of eighteen target polycyclic aromatic hydrocarbons in ambient aerosol. Although similar results were obtained in terms of both detection and quantitation limits, a larger number of analytes were identified by using the GC × GC-TOFMS method, thus suggesting that comprehensive two-dimensional gas-chromatography coupled to mass spectrometry such as GC × GC-TOFMS could be applicable to atmospheric and related sciences with simultaneous target and nontarget analyses in a single run.

Mass spectrometry can be considered the technology of the future for medicine: its capabilities in biomarker discovery, development, and validation suggest the implementation of MS instruments in clinical labs. Nowadays, MS-based lab detection methods are increasingly used in hospital labs as well as in legal medicine: in this context, R. Hösli and coworkers have compared the analytical performances of a GC-MS and a LC-MS/MS method, respectively, for the determination of phenytoin in different body compartments, i.e., blood, saliva, and human brain dialysate. The LC-MS/MS method proved to be more sensitive than the GC-MS procedure, being also less time-consuming and requiring small amount of sample. Finally, L. Huang et al. have reported the role of trichloroacetic acid in enhancing the MS signal of tobramycin. Using a simple dilution with trichloroacetic acid as pairing reagent, a sensitive LC-MS/MS method was developed and validated in a bacterial medium.

